# DNA aneuploidy with image cytometry for detecting dysplasia and carcinoma in oral potentially malignant disorders: A prospective diagnostic study

**DOI:** 10.1002/cam4.3293

**Published:** 2020-07-07

**Authors:** Chenxi Li, Lan Wu, Yiwen Deng, Xuemin Shen, Wei Liu, Linjun Shi

**Affiliations:** ^1^ Department of Oral Mucosal Diseases Shanghai Ninth People's Hospital College of Stomatology Shanghai Jiao Tong University School of Medicine National Clinical Research Center for Oral Diseases Shanghai Key Laboratory of Stomatology & Shanghai Research Institute of Stomatology Shanghai China; ^2^ Department of Oral and Maxillofacial‐Head and Neck Oncology Shanghai Ninth People's Hospital College of Stomatology Shanghai Jiao Tong University School of Medicine Shanghai China

**Keywords:** aneuploidy, DNA‐image cytometry, dysplasia, oral potentially malignant disorders, Oral squamous cell carcinoma

## Abstract

**Background:**

Current evidence on diagnostic value of aneuploidy with DNA image cytometry (ICM) using brushings for oral potentially malignant disorders (OPMD) is limited by sample size and inconsistent classification criteria of aneuploidy. This study aimed to explore the optimal cut‐off values of DNA content and evaluate the diagnostic accuracy of DNA‐ICM for detecting dysplasia and/or carcinoma in OPMD.

**Materials and Methods:**

A total of 401 consecutive patients with OPMD were enrolled in this prospective diagnostic study. Brushing and biopsy sample form each patient was processed by DNA‐ICM and histological examination respectively.

**Results:**

When the optimal cut‐off of at least one aneuploid cell with DNA index (DI) ≥2.3, the area under the curves (AUC) was 0.735 and positive predictive value was 92.7% for detecting dysplasia within OPMD. When the optimal cut‐off of at least one aneuploid cell with DI ≥ 3.5, the AUC was 0.851 and negative predictive values was 96.8% for detecting carcinoma within OPMD. Importantly, multivariate analysis revealed that aneuploidy with DI ≥ 2.3 in OPMD was significantly associated with dysplasia risk (adjusted OR, 5.52; 95%CI, 2.90‐10.51; *P* < .001), and aneuploidy with DI ≥ 3.5 in OPMD was strongly associated with malignant risk (adjusted OR, 21.05; 95%CI, 9.34‐47.41; *P* < .001).

**Conclusions:**

This largest‐scale diagnostic study optimized the criteria of aneuploidy cytology for noninvasive detection of oral dysplasia and carcinoma within OPMD. DNA aneuploidy in OPMD was an independent marker that strongly associated with oral dysplasia/carcinoma. Our findings suggest that DNA‐ICM may serve as a useful noninvasive adjunctive tool for oral cancer and OPMD screening.

## Graphical abstract

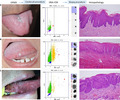

## 1 INTRODUCTION

Oral squamous cell carcinoma (OSCC) is often diagnosed at a late stage and therefore early detection and timely intervention are crucial for decreasing morbidity and mortality of OSCC.^1^, ^2^ A portion of OSCC preceded by oral potentially malignant disorders (OPMD), mainly being leukoplakia, and presence of dysplasia is often considered as a discriminator for malignant potential of OPMD.^3^, ^4^, ^5^ Currently, tissue biopsy and histological examination remain the standard for diagnosis of dysplasia and OSCC. Nevertheless, histologic assessment is known to be insufficient and may involve subjectivity.^4^, ^5^ It is doubtful whether an incisional biopsy sample from suspicious lesion is reliable and representative of the histological findings of the whole lesion.^5^ Besides, it is evident that invasive sequential biopsies have a limited reproducibility for the surveillance of patients with oral suspicious lesions. Therefore, adjunctive objective diagnostic techniques are required to early detect dysplasia and carcinoma within OPMD and contribute to surveillance of OPMD progression.^6^

Aneuploidy is a cancer‐type‐specific oncogenic event that may have clinical relevance as a prognostic marker and as a potential therapeutic target.^7^ DNA ploidy status determined by image cytometry (ICM) using brushings is an objective and noninvasive adjunctive diagnostic technique to automatically measure nuclear DNA content.^8^ Although DNA aneuploidy has been known to be a marker of malignancy in several organs including oral cavity, there is poor evidence so far of DNA aneuploidy cytology using brushings being successful as an adjunctive tool for oral cancer detection.^9^, ^10^ Particularly, current evidence must be interpreted prudently due to some reasons: small sample size, heterogeneity of the enrolled subjects, as well as, in particular, different classification criteria of DNA aneuploidy.^10^ Hence, more well‐designed studies are required to evaluate the diagnostic value of DNA‐ICM using brushings for OPMD and early OSCC.

It was previously reported that DNA‐ICM added in the diagnosis of high‐grade dysplasia and staging of oral leukoplakia in the small case series.^11^, ^12^ In view of the aforementioned limitations, the aim of this study, thus, was to explore the optimal cut‐off values of DNA content and evaluate the diagnostic accuracy of DNA‐ICM using brushings for detecting dysplasia and/or carcinoma in a large prospective series of OPMD patients. Notably, we further tested whether DNA aneuploidy was an independent discriminator for dysplasia and carcinoma combined with risk factors by logistic regression in the study.

## 2 MATERIALS AND METHODS

### 2.1 Patients and cytobrush procedure

This study was approved by our local Institutional Review Board and was registered in Chinese Clinical Trial Registry with written informed consent obtained from all participating subjects. In this clinic‐setting study, patients with clinical aspect of the OPMD lesion (oral leukoplakia, erythroplakia, oral submucous fibrosis (OSF), and oral lichenoid lesion (OLL)) who visited the clinic at the Department of Oral Mucosal Diseases of our hospital were prospectively enrolled during March 2013 to August 2018 period.

Before scalpel biopsy of the lesion was performed, each participant underwent cytobrush biopsy at the same location of the lesion. Brush sample was collected by a wide‐spread brushing of the whole lesion based on a liquid‐based brush kit (MotiSavant, Motic Inc, Xiamen, China). Next, scalpel biopsy was then taken from the same location of the brushing. The biopsies were fixed in formalin, embedded in paraffin, and processed for routine histopathologic examination at the Department of Oral Pathology of our hospital. Histological diagnosis was performed by two oral pathologists blinded to the DNA content results, according to the definition and classification described previously.^5^, ^13^, ^14^, ^15^

Leukoplakia is defined as a predominantly white lesion of the oral mucosa that cannot be characterized as any other definable lesion; A definitive diagnosis of leukoplakia is made when any etiological cause other than tobacco/areca nut use has been excluded and histopathology has not confirmed any other specific disorder.^5^ Erythroplakia is defined as a fiery red patch that cannot be characterized clinically or pathologically as any other definable disease; Numerous other red patches/macules that could arise on the oral mucosa should be excluded before considering erythroplakia as the diagnosis.^5^ Modified WHO diagnostic criteria of OLL proposed by van der Meij and van der Waal^13^ was used in this study (Table S1). OSF is defined as a chronic disorder characterized by fibrosis of the lining mucosa of entire oral cavity and sometimes pharynx.^15^

The inclusion and exclusion criteria are as follows:

Inclusion criteria: (a) Primary diagnosis of OPMD or OPMD concomitant suspicious OSCC. (b) Both brushing and biopsy samples were obtained. (c) DNA content analysis was completed by DNA‐ICM.

Exclusion criteria: (a) Primary diagnosis of OSCC without the history of OPMD. (b) Patient with the history of malignancy. (c) Specifically, patients with the clinical and pathological diagnosis of oral lichen planus (OLP) were excluded in this study due to its extremely low rate of DNA aneuploidy^16^ and the diagnosis of OLP being the absence of epithelial dysplasia.^13^, ^14^ Consistently, subjects diagnosed as OLP were also excluded in the previous relevant study on OPMD.^17^

### 2.2 DNA‐ICM analysis

DNA content status was analyzed using ICM according to the previous studies^10^, ^11^ and the manufacturer's protocol (MotiSavant, Motic Inc, Xiamen, China). The inconsistent criteria of DNA aneuploidy using brushings in diagnosis of OPMD and OSCC in English‐language literature recently summarized.^10^ In the majority of the previous studies, outside DNA index (DI) of 1.8‐2.2 and 3.6‐4.4 and/or 9c events was defined as aneuploidy criteria; whilst over 4 or 5 cells with DI > 2.3 was defined as DNA aneuploidy criteria used by some other studies.^10^ Consequently, the current study thus addressed the optimal cut‐off values of DNA aneuploidy in detecting dysplasia and carcinoma within OPMD in a large prospective study using the PICO method^9^:

P (Population): Patients with OPMD.

I (Intervention): Quantification of DNA content by ICM using brushings collected from the lesions.

**FIGURE 1:**
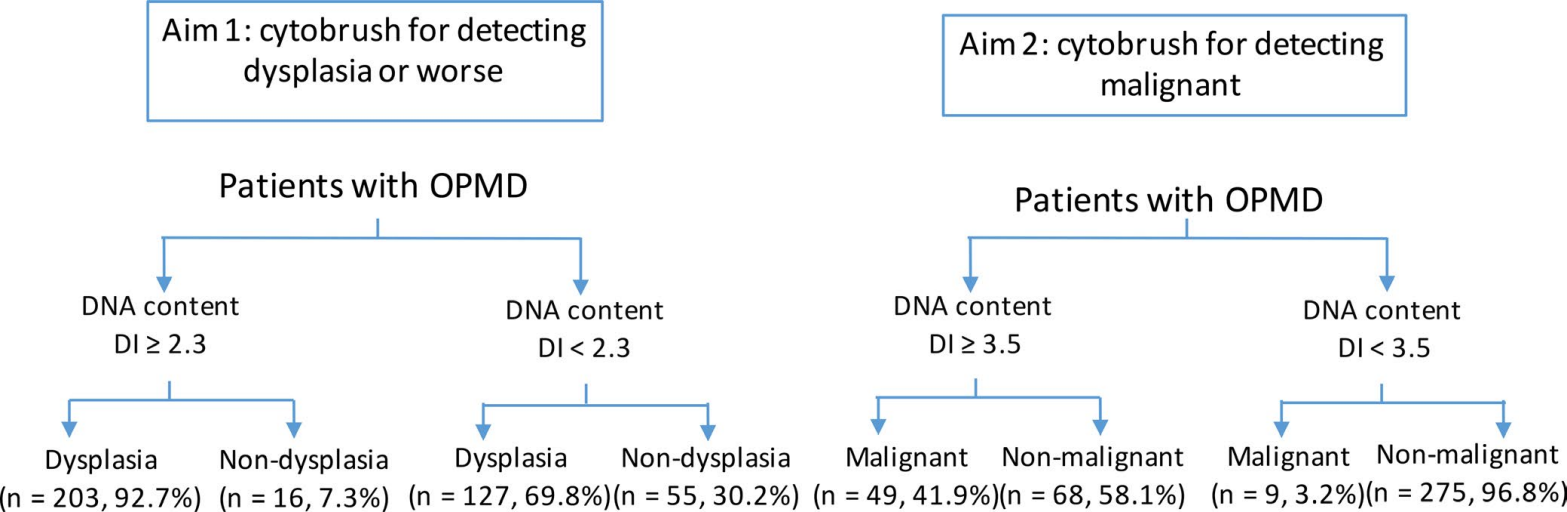
The flowchart of this study on oral potentially malignant disorders (OPMD)

C (Comparison): Histopathologic diagnosis of dysplasia and carcinoma.

(Outcome): (a) Discrimination of dysplasia or worse, hereinafter referred to as dysplasia, in general OPMD; (b) detection of carcinoma form general OPMD. The flowchart of this study was showed in Figure 1.

### 2.3 Statistical analysis

Differences in qualitative variables were calculated by the Fisher's exact test. Receiver operating characteristic (ROC) curves and the area under the curves (AUC) and its 95% confidence interval (CI) were constructed to evaluate the diagnostic values of DNA aneuploidy. Youden index, defined as the overall correct classification rate minus one at the optimal cut‐off point, is another important index. The optimal cut‐off thresholds were determined by using the maximum Youden index.^18^, ^19^ Statistics including sensitivity, specificity, positive and negative predictive values (PPV and NPV) with 95%CI were calculated to determine the diagnostic accuracy of aneuploidy. Logistic regression analysis was applied to evaluate odds ratio (OR) and the association among variables. In logistic regression, univariate analysis was first performed to obtain the significant variables. To further assess and adjust the influence of each significant variable, multivariate analysis was then performed to assess which factors remained statistical significance. Statistical analysis was performed using SPSS for Windows (version 21.0; SPSS Inc). All tests were two‐sided, and *P* values of < .05 were considered statistically significant.

## 3 RESULTS

### 3.1 Characteristics of enrolled patients with OPMD

**FIGURE 2:**
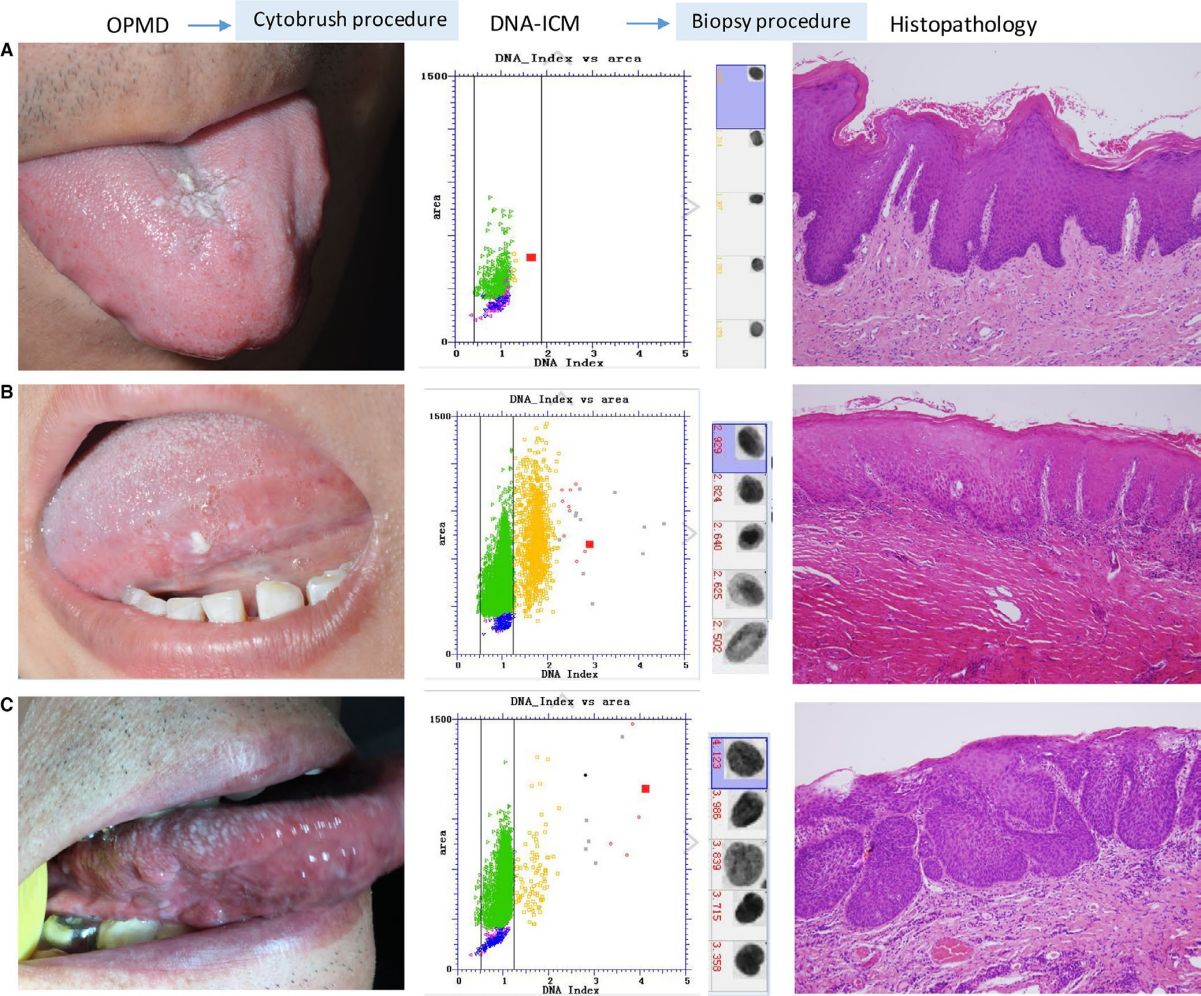
Representative clinical manifestation, DI values determined by DNA‐ICM, and histopathology of three cases of OPMD. A, A case of homogenous lesion with DI values < 2.3 was examined to be non‐dysplasia. B, A case of homogenous lesions with 2.3 < DI values < 3.5 was examined to be mild‐moderate dysplasia. C, A case of nonhomogenous lesion with DI values ≥ 3.5 was examined to be carcinoma within lesion

According to inclusion and exclusion criteria, a total of 401 consecutive patients with OPMD were enrolled in this prospective diagnostic study. There were 205 male and 196 female participants, and the average age was 53.2 years old (range, 18‐85 years). On subtype of OPMD, 316 (78.8%) of these patients were oral leukoplakia, followed by OLL (n = 44, 11.0%), OSF (n = 29, 7.2%), erythroplakia (n = 12, 3.0%). On histopathological assessment, 71 (17.7%) were with no dysplasia, 272 (67.8%) were with dysplasia, and the remaining 58 (14.5%) were with carcinoma. The history of smoking and alcohol intake were observed in 41.4% and 34.4% cases respectively. Representative clinical manifestation, DI values, and histopathology of three cases of OPMD were showed in Figure 2.

### 3.2 Optimization criteria of DNA aneuploidy for OPMD

**FIGURE 3:**
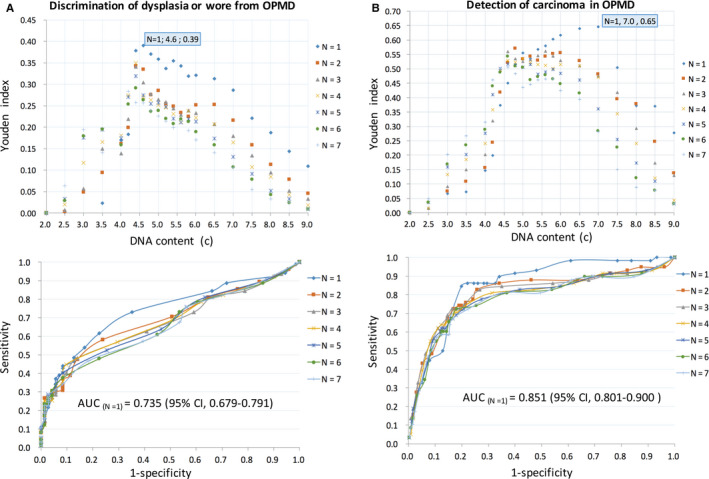
Diagnostic efficacy of DNA aneuploidy cytology in OPMD. A, Optimal cut‐off values of DNA content by using the maximum Youden index. B, ROC curve of the different number (N) of DNA content values

In the field of diagnostic studies, the AUC serves as an overall measure of a diagnostic test's accuracy. Optimal criterion for cut‐off point selection in the context of ROC curve analysis is the maximum of the Youden index.^18^, ^19^ To address the optimal cut‐off values of DNA aneuploidy cytology in detecting dysplasia and/or carcinoma within OPMD, ROC curve with AUC analysis was performed by using the highest Youden index (Figure 3). On comparison of the DNA‐ICM results with the histopathological diagnosis, the sensitivity, specificity, PPV, and NPV of the outcome assessment were presented in Table 1. For i) discrimination of dysplasia form OPMD, when the optimal cut‐off of at least one aneuploid cell with DI ≥ 2.3 (DNA content ≥ 4.6c), the AUC was 0.735 (sensitivity = 61.5%, specificity = 77.5%); ii) detection of carcinoma within OPMD, when the optimal cut‐off of at least one aneuploid cell with DI ≥ 3.5 (DNA content ≥ 7.0c), the AUC was 0.851 (sensitivity = 84.5%, specificity = 80.2%). Notably, the PPV of detecting dysplasia was 92.7%, and the NPV of detecting carcinoma was 96.8%.

**TABLE 1:** Sensitivity, specificity, positive, and negative predictive values (PPV and NPV) of DNA index for oral potentially malignant disorders (OPMD)

Criteria of DNA aneuploidy for detecting dysplasia or worse in OPMD			
DI value	One cell with DI ≥ 2.3	One cell with DI < 2.3	Statistic (95% CI)
Dysplasia or worse	True positive (n = 203)	False positive (n = 127)	Sensitivity = 61.5% (56.2%‐66.6%)
Nondysplasia	False negative (n = 16)	True negative (n = 55)	Specificity = 77.5% (66.4%‐85.7%)
			PPV = 92.7% (88.4%‐95.5%)
			NPV = 30.2% (24.0%‐37.3%)

Criteria of DNA aneuploidy for detecting carcinoma in OPMD			
DI value	One cell with DI ≥ 3.5	One cell with DI < 3.5	Statistic (95% CI)
Malignant	True positive (n = 49)	False positive (n = 9)	Sensitivity = 84.5% (72.8%‐91.8%)
Nonmalignant	False negative (n = 68)	True negative (n = 275)	Specificity = 80.2% (75.6%‐84.1%)
			PPV = 41.9% (33.3%‐50.9%)
			NPV = 96.8% (94.0%‐98.4%)

### 3.3 Odds ratio analysis of DNA aneuploidy criteria in OPMD

To further clarify the potential confounding variables, odds ratio analysis by logistic regression for assessing the association between DNA aneuploidy and demographic (age and gender) and genotoxic (smoking and alcohol intake) factors within OPMD. For (a) detecting dysplasia within OPMD (Table 2), multivariate analysis revealed that age (≥60 years) and alcohol drinking were significantly associated with dysplasia (both *P* < .05), whereas DNA aneuploidy with DI ≥ 2.3 in OPMD was strongly associated with dysplasia risk (adjusted OR, 5.52; 95% CI, 2.90‐10.51; *P* < .001). For (b) detecting carcinoma within OPMD (Table 3), multivariate analysis revealed that alcohol drinking was significantly associated with carcinoma (*P* = .014), whereas DNA aneuploidy with DI ≥ 3.5 in OPMD was greater associated with malignant risk (adjusted OR, 21.05; 95% CI, 9.34‐47.41; *P* < .001).

**TABLE 2:** Assessment of DNA aueuploidy criteria for detecting dysplasia/carcinoma in oral potentially malignant disorders (OPMD)

Characteristic	No dysplasia	Dysplasia or worse	Fisher's exact test	Univariate analysis	Multivariate analysis
Total, n (%)	71 (17.7)	330 (82.3)		OR (95%CI)	OR (95%CI)
Age (y)			*P* = .036	*P* = .030	*P* = .043
<60	60 (20.2)	237 (79.8)		1.0 (ref)	1.0 (ref)
≥60	11 (10.6)	93 (89.4)		2.14 (1.08‐4.25)	2.18 (1.02‐4.66)
Gender			*P* = .006	*P* = .006	*P* = .164
Female	24 (12.2)	172 (87.8)		1.0 (ref)	1.0 (ref)
Male	47 (22.9)	158 (77.1)		0.47 (0.27‐0.80)	0.55 (0.24‐1.27)
Cigarette smoking			*P* = .014	*P* = .014	*P* = .298
Never	27 (13.5)	173 (86.5)		1.0 (ref)	1.0 (ref)
Past/present	39 (23.5)	127 (76.5)		0.51 (0.30‐0.87)	1.62 (0.65‐4.03)
Alcohol drinking			*P* = .002	*P* = .002	*P* = .014
Never	30 (13.1)	199 (86.9)		1.0 (ref)	1.0 (ref)
Past/present	36 (26.3)	101 (73.7)		0.42 (0.25‐0.73)	0.37 (0.17‐0.82)
Criteria of DNA aneuploidy			*P* < .001	*P* < .001	*P* < .001
DI < 2.3	55 (30.2)	127 (69.8)		1.0 (ref)	1.0 (ref)
DI ≥ 2.3	16 (7.3)	203 (92.7)		5.50 (3.02‐10.00)	5.52 (2.90‐10.51)

**TABLE 3:** Assessment of DNA aueuploidy criteria for detecting carcinoma in oral potentially malignant disorders (OPMD)

Characteristic	Nonmalignant	Malignant	Fisher's exact test	Univariate analysis	Multivariate analysis
Total, n (%)	343 (85.5)	58 (14.5)		OR (95%CI)	OR (95%CI)
Age (y)			*P* = 1.000	*P* = .989	
<60	254 (85.5)	43 (14.5)		1.0 (ref)	
≥60	89 (85.6)	15 (14.4)		1.00 (0.53‐1.88)	
Gender			*P* = .480	*P* = .452	
Female	165 (84.2)	31 (15.8)		1.0 (ref)	
Male	178 (86.8)	27 (13.2)		0.81 (0.46‐1.41)	
Smoking			*P* = .093	*P* = .142	
Never	177 (88.5)	23 (11.5)		1.0 (ref)	
Past/present	138 (83.1)	28 (16.9)		1.56 (0.86‐2.83)	
Alcohol drinking			*P* = .008	*P* = .006	*P* = .012
Never	206 (90.0)	23 (10.0)		1.0 (ref)	1.0 (ref)
Past/present	109 (79.6)	28 (20.4)		2.30 (1.27‐4.19)	2.43 (1.22‐4.85)
Criteria of DNA aneuploidy			*P* < .001	*P* < .001	*P* < .001
DI < 3.5	275 (96.8)	9 (3.2)		1.0 (ref)	1.0 (ref)
DI ≥ 3.5	68 (58.1)	49 (41.9)		22.02 (10.31‐47.02)	21.05 (9.34‐47.41)

### 3.4 Pathologic diagnosis and DNA aneuploidy distribution by the subtypes of OPMD

The clinical subtypes of OPMD have a different malignant potential, the demographics, pathologic diagnosis, DI values distribution by the subtypes of OPMD including leukoplakia, OLL, OSF, erythroplakia were analyzed. As showed in Table 4, all the pathologic diagnosis of OSF were no/mild dysplasia, and the vast majority of OSF were then found to be normal DNA content. Conversely, 91.7% of erythroplakia were severe dysplasia and carcinoma, and the vast majority of erythroplakia were then found to be abnormal DNA content. The proportion (14.9%) of carcinoma within leukoplakia was higher than that (4.5%) of carcinoma within OLL. Consistently, the proportion (32.0%) of aneuploidy with DI ≥ 3.5 within leukoplakia was significantly higher than that (13.6%) of aneuploidy with DI ≥ 3.5 within OLL (*P* = .013, Fisher's exact test).

**TABLE 4:** Baseline characteristics of demographics, pathologic diagnosis, DI values by the subtypes of OPMD

Characteristic	Leukoplakia	Lichenoid lesion		Submucous fibrosis		Erythroplakia	
Total, n (%)	316	44		29		12	
Age (y)							
<60	235 (74.4)	33 (75.0)		19 (65.5)		10 (83.3)	
≥60	81 (25.6)	11 (25.0)		10 (34.5)		2 (16.7)	
Gender							
Female	162 (51.3)	23 (52.3)		0		11 (91.7)	
Male	154 (48.7)	21 (47.7)		29 (100)		1 (8.3)	
Pathologic diagnosis							
No dysplasia	39 (12.3)	11 (25.0)		21 (72.4)		0	
Mild dysplasia	134 (42.4)	22 (50.0)		8 (27.6)		1 (8.3)	
Moderate dysplasia	79 (25.0)	7 (15.9)		0		0	
Severe dysplasia	17 (5.4)	2 (4.5)		0		2 (16.7)	
Carcinoma	47 (14.9)	2 (4.5)		0		9 (75.0)	
DNA aneuploidy for dysplasia							
DI < 2.3	133 (42.1)	21 (47.7)		26 (89.7)		2 (16.7)	
DI ≥ 2.3	183 (57.9)	23 (52.3)		3 (10.3)		10 (83.3)	
DNA aneuploidy for carcinoma							
DI < 3.5	215 (68.0)	38 (86.4)		28 (96.6)		3 (25.0)	
DI ≥ 3.5	101 (32.0)	6 (13.6)		1 (3.4)		9 (75.0)	

## 4 DISCUSSION

DNA aneuploidy is an indicator of numerical chromosomal changes and its emergence is usually an early crucial step in carcinogenesis,^20^ despite lately its reputation as a marker of oral cancer progression was ever questioned.^21^, ^22^ For formalin fixed paraffin‐embedded biopsy tissues, DNA aneuploidy is reported to be a useful marker in predicting malignant transformation of OPMD by meta‐analysis.^23^ DNA aneuploidy cytology using oral brushings is a potential noninvasive adjunctive diagnostic tool in early detection of oral cancer, but current evidence is limited mainly by small sample size, heterogeneity of the enrolled subjects, and different classification criteria of aneuploidy of previous studies.^8^, ^9^ To the best of our knowledge, the sample size (n = 401) of the current study was the largest‐scale series in a single study on the diagnostic value of the DNA‐ICM using brushings for detection of oral carcinoma and dysplasia within OPMD with homogeneity of the enrolled subjects.

Previous studies reported a wide range of sensitivity (16.0‐96.4%) and specificity (66.6‐100%) of DNA‐ICM in screening OPMD using brushings (reviewed in Ref. [9]), due to the differences in study design and criteria of aneuploidy.^9^, ^10^ Difference in the number and proportion of studied subjects may produce different results. The sample size in the majority of the previous studies was not large,^9^, ^10^ since sample size is important to cancer screening. Notably, the various proportions of OSCC, dysplasia, and benign lesions enrolled in the study could produce different results, since detection of OSCC or dysplasia is the exposed factor. Conceivably, the higher proportions of OSCC and benign lesions and lower proportion of OPMD would increase the diagnostic sensitivity and specificity in screening OPMD.^10^ Arguably, the proportion (14.5%) of patients with concomitant OSCC/focal cancerization in this study was reasonable, compared to high proportions (22.0%‐50.0%) of OSCC in previous studies (summarized in ref.10). Moreover, the proportion (14.5%) was within the rate (12.2%‐17.9%) of malignant transformation of oral leukoplakia from China.^24^, ^25^

Although it was moderate efficacy that that AUC of DNA aneuploidy was 0.735 (sensitivity, 61.5%; specificity, 77.5%) for discrimination of dysplasia from general OPMD, it was well‐recognized efficacy that the AUC of DNA aneuploidy was 0.851 (sensitivity, 84.5%; specificity, 80.2%) for detection of carcinoma in general OPMD. Correspondingly, the optimal criteria of DNA aneuploidy were classified from at least one aneuploid cell with DI ≥ 2.3 to aneuploid cell with DI ≥ 3.5. Moreover, DNA aneuploidy using image cytometry represented an early event and may serve as an independent marker that strongly associated with oral dysplasia and carcinoma, in agreement with the aneuploidy results analyzed by flow cytometry.^17^ Of particular interest, the presence of DNA aneuploidy in inflammatory bowel disease determined by flow cytometry was also associated with development of dysplasia or colorectal cancer.^26^ Our profile of DNA aneuploidy to some extent reflected that the malignant progression of OPMD is a typical multistep carcinogenesis processes with stepwise accumulations of DNA and genetic alterations. These findings suggest that DNA aneuploidy using brushing sample could be used as an indicator of disease before the appearance of clinical signs and symptoms in early OSCC patients.

In the current study, we found that the high PPV (92.7%) of detecting dysplasia indicates that to the OPMD patients would be more recommend invasive biopsy to diagnose whether dysplasia within OPMD when DI ≥ 2.3 determined by DNA‐ICM. This may suggest that DNA‐ICM have the potential application as a screening tool for the detection of dysplastic OPMD in general dental and community practice. Furthermore, the NPV (96.8%) of detecting carcinoma indicates that the probability of OPMD patients with DI < 3.5 being OSCC would be relatively low. DI < 3.5 thus may reduce the need for invasive sequential biopsies and reduce the patients’ psychological burden of cancerization due to the high NPV. If tumor cells or DI ≥ 3.5 are present, a total surgical excision in favor of a biopsy of the respective lesion and histological examination should be strongly considered. If DI < 3.5 is present and combined with clinical oral examination, a wait‐and‐watch strategy for patients with not DNA aneuploid lesions may be appropriate. Although some molecular biomarkers were reported to have the prognostic value of oral dysplasia progressed to carcinoma,^27^ DNA‐ICM may have clinical applications in the future as a surveillance tool for noninvasive detection of oral cancer in specialist clinical practice.

We are aware of the limitations of this study that the examination itself is of some skill and training are required while interpreting the DNA‐ICM procedure. Besides, the potential practice of this study is an oral medicine specialist practitioner setting, and further studies should evaluate the practicability of this procedure in general dental and community screening. The research combined DNA‐ICM with other noninvasive techniques like optical fluorescence, microRNA, human papillomavirus leading to improved test results are warranted.^28^, ^29^, ^30^ It should be noted that the vast majority of the study subjects were patients with leukoplakia and the subtypes of the OPMD were very poorly balanced; therefore, the analysis of the other OPMDs separately was underpowered. Moreover, this investigation was a cross‐section diagnostic study. We also realize that longitudinal studies with adequate follow‐up and clinical endpoints are required to evaluate the efficacy of the DNA aneuploidy cytology as a surveillance tool for oral cancer development. The longitudinal studies combined DNA aneuploidy with other predictive biomarkers including SMAD4 and CCND1 alterations are warranted.^27^, ^31^, ^32^ Meanwhile, the poor NPV (30.2%) of detecting dysplasia and the PPV (41.9%) of detecting carcinoma within OPMD were the limitations of DNA‐ICM in future studies. Besides, it was moderate or plain efficacy of DNA aneuploidy for diagnosing dysplasia in OPMD excluding carcinoma of histopathology (Figure S1).

## 5 CONCLUSIONS

This largest‐scale diagnostic study optimized the criteria of aneuploidy with DNA‐ICM for noninvasive detection of oral dysplasia (DI ≥ 2.3) and carcinoma (DI ≥ 3.5) in a large prospective series of OPMD patients from China. Notably, we demonstrated that DNA aneuploidy in OPMD was an independent discriminator for oral dysplasia and carcinoma by logistic regression. Our findings indicate DNA‐ICM using brushings may be a promising tool for noninvasive detection of dysplasia and carcinoma within OPMD. Longitudinal studies on DNA‐ICM using oral brushing sample collection at different times during follow‐up as a surveillance tool for oral cancer progression are warranted.

## CONFLICTS OF INTEREST

None.

## AUTHORS' CONTRIBUTIONS

CL and LW performed the experiments, analyzed the data, and wrote the manuscript. YD and XS contributed to the sample acquisition and the manuscript revision. LS and WL conceived and designed the study and performed data interpretation. All authors have reviewed and approved the manuscript.

## Supporting information

Fig S1

Table S1

## Data Availability

The data that support the findings of this study are available from the corresponding author upon reasonable request.
