# Supplementary figures and images for: DNA aneuploidy with image cytometry for detecting dysplasia and carcinoma in oral potentially malignant disorders: A prospective diagnostic study
Source: Cancer Med. 2020 Jul 7;9(17):6411–20. doi: 10.1002/cam4.3293 (PMC7476813; doi:10.1002/cam4.3293)

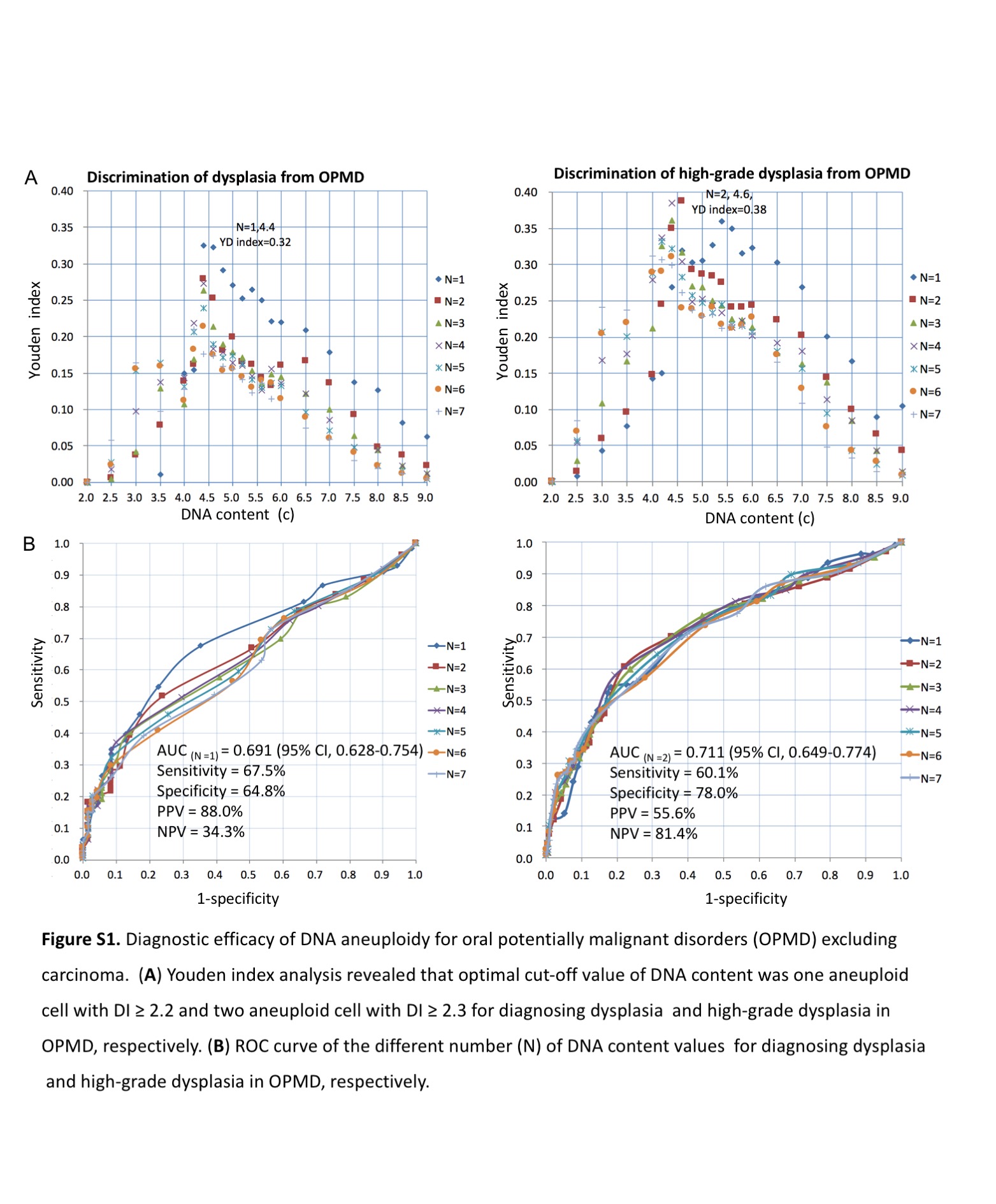

Supplement: Supplementary file 1 — Fig S1 [file CAM4-9-6411-s001.jpg]
