# Supplementary material for: DNA aneuploidy with image cytometry for detecting dysplasia and carcinoma in oral potentially malignant disorders: A prospective diagnostic study
Source: Cancer Med. 2020 Jul 7;9(17):6411–20. doi: 10.1002/cam4.3293 (PMC7476813; doi:10.1002/cam4.3293)
Supplement: Supplementary file 2 — Table S1 [file CAM4-9-6411-s002.docx]

Table S1. Modified WHO diagnostic criteria of oral lichen planus (OLP) and oral lichenoid lesions (OLL) proposed by van der Meij and van der Waal

| **Clinical criteria** | |
| --- | --- |
| Presence of bilateral, more or less symmetrical lesions | |
| Presence of a lace-like network of slightly raised gray-white lines (reticular pattern) | |
| Erosive, atrophic, bulbous and plaque-type lesions are only accepted as a subtype in the presence of reticular lesions elsewhere in the oral mucosa In all other lesions that resemble OLP but do not complete the aforementioned criteria, the term `clinically compatible with' should be used | |
| **Histopathologic criteria** | |
| Presence of a well-defined band-like zone of cellular infiltration that is confined to the superficial part of the connective tissue, consisting mainly of lymphocytes Signs of `liquefaction degeneration' in the basal cell layer | |
| Absence of epithelial dysplasia | |
| When the histopathologic features are less obvious, the term `histopathologically compatible with' should be used | |
| **Final diagnosis OLP or OLL** | |
| To achieve a final diagnosis clinical as well as histopathologic criteria should be included | |
| OLP | A diagnosis of OLP requires fulfillment of both clinical and histopathologic criteria |
| OLL | The term OLL will be used under the following conditions: |
|  | 1. Clinically typical of OLP but histopathologically only `compatible with' OLP |
|  | 2. Histopathologically typical of OLP but clinically only `compatible with' OLP |
|  | 3. Clinically `compatible with' OLP and histopathologically `compatible with' OLP |
